# Preoperative plasma fibrinogen and C-reactive protein/albumin ratio as prognostic biomarkers for pancreatic carcinoma

**DOI:** 10.3389/fonc.2024.1301059

**Published:** 2024-03-01

**Authors:** Xiaopeng Chen, Zhaohui Chen, Jianyang Guo, Zhe Xiu, Huangxiang Chen

**Affiliations:** ^1^ Department of Hepatobiliary Surgery, The Second Hospital of Longyan, Longyan, China; ^2^ Department of the 9th Affiliated Hospital of Xi'an Jiaotong University, Xian, China

**Keywords:** plasma fibrinogen, C-reactive protein/albumin, overall survival, prognosis, pancreatic carcinoma

## Abstract

**Objective:**

Pancreatic carcinoma is characterised by high aggressiveness and a bleak prognosis; optimising related treatment decisions depends on the availability of reliable prognostic markers. This study was designed to compare various blood biomarkers, such as neutrophil/lymphocyte ratio (NLR), lymphocyte/monocyte ratio (LMR), platelet/lymphocyte ratio (PLR), C-reactive protein (CRP), albumin (Alb), plasma fibrinogen (PF), and CRP/Alb in patients with pancreatic carcinoma.

**Methods:**

Our study retrospectively reviewed 250 patients with pancreatic carcinoma diagnosed between July 2007 and December 2018. The Cutoff Finder application was used to calculate the optimal values of CRP/Alb and PF. The Chi-square test or Fisher’s exact test was used to analyse the correlation of CRP/Alb and PF with other clinicopathological factors. Conducting univariate and multivariate analyses allowed further survival analysis of these prognostic factors.

**Results:**

Multivariate analysis revealed that, in a cohort of 232 patients with pancreatic ductal adenocarcinoma (PDAC), the PF level exhibited statistical significance for overall survival (hazard ratio (HR) = 0.464; *p* = 0.023); however, this correlation was not found in the entire group of 250 patients with pancreatic carcinoma. Contrastingly, the CRP/Alb ratio was demonstrated statistical significance in both the entire pancreatic carcinoma cohort (HR = 0.471; *p* = 0.026) and the PDAC subgroup (HR = 0.484; *p* = 0.034). CRP/Alb and PF demonstrated a positive association (r=0.489, p<0.001) as indicated by Spearman’s rank correlation analysis. Additionally, in 232 PDAC patients, the combination of the CRP/Alb ratio and PF had synergistic effects on prognosis when compared with either the CRP/Alb ratio or the PF concentration alone.

**Conclusion:**

PF concentration is a convenient, rapid, and noninvasive biomarker, and its combination with the CRP/Alb ratio could significantly enhance the accuracy of prognosis prediction in pancreatic carcinoma patients, especially those with the most common histological subtype of PDAC.

## 1 Introduction

Pancreatic carcinoma (PC) is a malignant tumour arising from pancreatic cells. The pancreas is an abdominal glandular organ essential for food digestion and blood sugar level regulation (1, 2). It accounts for 216,000 new cancer cases each year, resulting in more than 200,000 annual deaths worldwide (3, 4). Pancreatic carcinoma is a highly aggressive form of cancer and is typically diagnosed in advanced stages when the tumour has already spread to other organs. Pancreatic ductal adenocarcinoma (PDAC), the most prevalent form of pancreatic carcinoma, constitutes nearly 95% of all pancreatic carcinoma cases (5). Symptoms of pancreatic carcinoma include abdominal pain, unexplained weight loss, appetite loss, nausea, vomiting, jaundice, and alterations in stool colour. Therapeutic approaches for pancreatic carcinoma may include surgical intervention, chemotherapy, radiation therapy, or a combination thereof, with selection dependent on factors such as tumour stage, location, and overall health (6). While these treatments have significantly enhanced the survival prospects for individuals with pancreatic carcinoma over the past few years, the survival status of patients in this situation continues to be grim, with a five-year survival rate typically falling below 10% (7).

The role of several prognostic indicators, such as the C-reactive protein/albumin (CRP/Alb) ratio and neutrophil/lymphocyte ratio (NLR), in the occurrence and development of pancreatic carcinoma has been confirmed (8, 9). Nevertheless, thus far, most of these markers have not been used in the general clinical setting due to a lack of therapeutic effectiveness in pancreatic carcinoma patients. Therefore, identifying novel reliable biomarkers for the application of prognostic factors in pancreatic carcinoma is essential, especially for enhanced risk stratification and more individualised clinical treatment (10).

The potential usefulness of the CRP/Alb as a biomarker has been highlighted in various tumours, such as digestive system tumours and gynaecological carcinomas (11–13). However, the potential of the preoperative CRP/Alb to function as a prognostic indicator in individuals with pancreatic carcinoma and whether its prognostic efficacy surpasses that of other inflammatory prognostic factors are unclear.

Previous studies have suggested that various indicators from the coagulation/fibrinolysis system, especially plasma fibrinogen (PF) and D-dimer levels, are abnormal in cancer patients (14–16). PF is a 340-kDa plasma glycoprotein that is key in the maintenance of haemostasis and participates in both inflammatory mechanisms and tumorigenesis (17). Numerous studies have confirmed that an increase in PF may promote the occurrence and development of various tumours (18–20). However, research examining its prognostic relevance in pancreatic carcinoma is limited.

Hence, our objective was to assess the ability of PF to predict oncological outcomes in pancreatic carcinoma patients in comparison to other inflammation-related metrics (albumin, CRP, and CRP/Alb) as well as cellular indicators (the NLR, lymphocyte/monocyte ratio (LMR), and platelet/lymphocyte ratio (PLR).

## 2 Methods

### 2.1 Patients

Between July 1, 2007, and December 1, 2018, 297 patient samples were newly diagnosed as showing pancreatic carcinoma at Longyan Second People’s Hospital and the 9th Affiliated Hospital of Xi’an Jiaotong University. The Medical Ethics Committee of the Second People’s Hospital of Longyan City has conducted a thorough review of medical records for all patients and approved their usage. The inclusion criteria were summarised as follows: (1) Chinese individuals who underwent radical surgery for primary pancreatic carcinoma and axillary lymph node dissection and (2) who had adequate and useful clinical data in their medical records. Among the exclusion criteria were the following: 1) patients with concurrent liver diseases, autoimmune diseases or coagulopathies requiring anticoagulants (n =15); 2) patients who had distant metastases or other malignancies at the time of diagnosis (n = 9); 3) patients receiving corticosteroids, oral contraceptives or hormone replacement therapy (n = 7); 4) patients who had received neoadjuvant therapy within 3 months (n = 8); and 5) patients with insufficient or invalid medical clinical data (n = 8). Ultimately, following application of the inclusion and exclusion criteria, a retrospective study was conducted involving 250 patients diagnosed with pancreatic carcinoma.

The clinical information gathered from the patient medical records included age, size, histological subtype, grade, lymph node metastasis (LNM) involvement, chemoradiotherapy data, neoadjuvant therapy data, and outcome data. Histopathological examination by two different pathologists confirmed the diagnosis of pancreatic carcinoma in all the samples. The PDAC patients included in the study were all pathologically confirmed and staged according to the 8th edition of the UICC TNM classification.

Pancreatic carcinoma patients underwent regular monitoring through telephone check-ins or postoperative appointments. Overall survival (OS) was characterised as the duration from the surgical procedure to the most recent follow-up visit, which was conducted on December 1, 2023, or deceased due to various reasons. The median duration of follow-up was 38 months, with a range spanning from 2 to 97 months.

### 2.2 Blood collection and assay methods

The plasma fibrinogen and CRP/Alb concentrations were measured in peripheral venous blood samples collected before breakfast less than 7 days before the start of surgery. Plasma was collected in a 5 ml blood collection tube and processed within 24 hours to measure the plasma coagulation parameters. The plasma fibrinogen levels were measured using the clotting method of Clauss. Using an automatic biochemical analyser (Hitachi 7600, Tokyo, Japan), we measured the CRP and albumin levels. With respect to all pancreatic carcinoma specimens, we used the ACL TOP system (Instrumentation Laboratory, Milan, Italy) to measure the plasma fibrinogen concentration. Data on white blood cells, neutrophils, lymphocytes, and platelet counts were collected with an automated haematology system (Sysmex XE-5000, Kobe, Japan).

### 2.3 Optimal prognostic cutoff values for inflammatory parameters

The determination of the optimal cutoff value followed the approach described by Gui et al. in 2021, utilising the minimum p value method (21). According to the prognostic scoring system, the optimal cutoff value for preoperative PF was 3.28 g/L, and for the CRP/Alb ratio, it was 0.18 (**Supplementary Figure 1**). The corresponding areas under the ROC curve for PF and the CRP/Alb ratio, representing their maximum values, were 0.679 and 0.803, respectively. The samples that were incorporated were split into two categories, where the low-level group comprised values below the optimal cutoff threshold and the high-level group consisted of values surpassing the optimal cutoff threshold.

Out of the 250 patients, the median PF was 3.53 g/L, and the mean PF was 3.31 g/L. These 250 patients were stratified into different groups based on their NLR, LMR, PLR, CRP, and albumin levels utilising the optimised cutoff values obtained from receiver operating characteristic (ROC) curve analysis; these cutoff values were defined as >3.10 mg/L, <3.06 mg/L, >128 mg/L, >5.1 mg/L, and <3.2 g/dL, respectively, to predict OS.

### 2.4 Statistical analysis

The software used for statistical analysis was SPSS 26.0 (IBM, Armonk, NY, USA). ROC curves were generated to predict 5-year OS and identify the optimal cutoff threshold for coagulation parameters, leading to the use of binary variables as treatment variables. The Chi-square test was applied to analyse the correlations between PF and the CRP/Alb ratio and clinicopathological parameters. The Kaplan−Meier method was used to construct survival curves, and comparisons were conducted using the log-rank test. To evaluate the difference in OS between the high-PF and CRP/Alb groups and between the low-PF and CRP/Alb ratio groups, univariate and multivariate Cox proportional hazards models were employed. Values where p < 0.05 were regarded as statistically significant.

## 3 Results

### 3.1 Patients’ features

From July 2007 to December 2018, 250 patients with pancreatic carcinoma who satisfied all the inclusion criteria were selected (**Table 1**). The median age at diagnosis was 53.6 years, (ranging, 29-81 years), and consisted of 145 males and 105 females. The 5-year OS rate among the 250 pancreatic carcinoma patients reached 15.8%. Overall, 104 patients exhibited good-to-moderate tumour differentiation, while 146 patients exhibited poor tumour differentiation. According to the 8th edition of the UICC, there were 62 patients with TNM stage T1 tumours and 50 patients with T2 tumours. T3 stage disease exhibited the highest incidence (80 cases), followed by the T4 stage (58 cases). One hundred and four patients (41.6%) had tumours < 2 cm, and 146 (58.4%) patients had tumours ≥ 2 cm. Out of a total of 250 patients, 149 had lymph node metastasis, while 101 did not. Detailed information regarding the treatment attributes, clinical profiles, and histopathological features of these patients is available in **Table 1**.

**Table 1:** Univariate and multivariate analyses of characteristics associated with OS in all 250 pancreatic carcinoma patients.

Characteristics	Univariate			Multivariate		
	Hazard Ratio	95%CI	*P-value*	Hazard Ratio	95%CI	*P-value*
Age, years						
*≥*50 *vs <*50	1.007	0.733-1.382	0.967	0.924	0.665-1.285	0.638
Gender						
Male *vs* Female	0.863	0.635-1.173	0.347	0.846	0.617-1.158	0.296
Clinical T stage						
>T2 *vs* ≤T2	2.615	1.875-3.647	<0.001	1.513	1.059-2.163	0.023
Tumour differentiation						
Poorly *vs* Well/moderately	1.808	1.307-2.501	<0.001	1.745	1.248-2.440	0.001
Tumour size						
≥2cm *vs <*2cm	1.032	0.755-1.410	0.844	0.992	0.721-1.366	0.962
LNM						
Yes *vs* No	2.209	1.564-3.120	<0.001	1.459	1.014-2.100	0.042
PF						
≥3.28 g/L *vs <*3.28 g/L	2.384	1.655-3.434	<0.001	0.568	0.303-1.064	0.078
NLR						
≥3.10 *vs <*3.10	1.384	1.001-1.915	0.046	1.272	0.906-1.786	0.165
LMR						
<3.06 *vs* ≥3.06	1.072	0.785-1.465	0.661	1.055	0.751-1.483	0.757
PLR						
≥128 *vs <*128	1.022	0.750-1.393	0.892	1.096	0.796-1.507	0.575
CRP						
≥5.1 mg/L *vs <*5.1mg/L	1.385	1.010-1.899	0.043	1.354	0.2326-1.859	0.061
Serum albumin						
<3.2g/dL *vs*≥3.2g/dL	1.245	0.913-1.696	0.166	1.093	0.792-1.508	0.587
CRP/Alb						
> 0.18 *vs* ≤ 0.18	2.318	1.625-3.305	<0.001	0.471	0.243-0.914	0.026
PF+ CRP/Alb						
PF-high+ CRP/Alb-high *vs* PF-high or CRP/Alb-high *vs* PF-low+ CRP/Alb-low	2.662	1.704-4.160	<0.001	8.034	3.410-18.932	<0.001

P-values that achieved statistical significance (p < 0.05) are indicated in bold. Alb, albumin; CRP, C-reactive protein; LMR, lymphocyte-monocyte ratio; LNM, lymph node metastasis; NLR, neutrophil-lymphocyte ratio; PF, plasma fibrinogen; PLR, platelet-lymphocyte ratio.

### 3.2 High PF and CRP/Alb and OS in pancreatic carcinoma patients

To assess the prognostic importance of PF and other prognostic parameters, we constructed a Cox proportional hazard regression model for both the pancreatic carcinoma subgroup and the PDAC subgroup. Within the univariate analysis of OS in the 250 patients, differences in several variables, including tumour differentiation (HR = 1.808; *p <*0.001), LNM (HR = 2.209; *p* < 0.001), clinical T stage (HR = 2.615; *p* < 0.001), PF (HR = 2.384; *p* < 0.001), NLR (HR = 1.384; *p* = 0.046), CRP (HR = 1.385; *p* = 0.043), and CRP/Alb (HR = 2.318; *p* < 0.001), exhibited statistical significance (**Table 1**). However, in the subgroup consisting of 232 PDAC samples, there was no significant difference in CRP levels (*p* = 0.914) (**Table 2**).

**Table 2:** Univariate and multivariate analyses of characteristics associated with OS in 232 PDAC patients.

Characteristics	Univariate			Multivariate		
	Hazard Ratio	95%CI	*P-*value	Hazard Ratio	95%CI	*P-*value
Age, years						
*≥*50 *vs <*50	1.033	0.744-1.436	0.846	0.954	0.674-1.349	0.789
Gender						
Male *vs* Female	0.885	0.644-1.216	0.451	0.877	0.633-1.214	0.429
Clinical T stage						
>T2 *vs* ≤T2	2.664	1.889-3.756	<0.001	1.510	1.036-2.201	0.032
Tumour differentiation						
Poorly *vs* Well/moderately	1.873	1.340-2.619	<0.001	1.796	1.270-2.540	<0.001
Tumour size						
≥2cm *vs <*2cm	1.044	0.755-1.444	0.796	1.078	0.775-1.499	0.655
LNM						
Yes *vs* No	2.417	1.676-3.487	<0.001	1.483	1.005-2.189	0.047
PF						
≥3.28 g/L *vs <*3.28 g/L	2.121	1.462-3.078	<0.001	0.464	0.239-0.899	0.023
NLR						
≥3.10 *vs <*3.10	1.424	1.022-1.2323	0.037	1.388	0.991-1.945	0.057
LMR						
<3.06 *vs* ≥3.06	1.012	0.734-1.396	0.942	0.971	0.683-1.379	0.868
PLR						
≥128 *vs <*128	1.027	0.746-1.414	0.872	1.109	0.799-1.538	0.538
CRP						
≥5.1 mg/L *vs <*5.1 mg/L	1.018	0.737-1.406	0.914	1.093	0.776-1.539	0.613
Serum albumin						
<3.5g/dL *vs* ≥3.5g/dL	1.320	0.956-1.821	0.092	1.137	0.808-1.600	0.462
CRP/Alb						
> 0.18 *vs* ≤ 0.18	2.449	1.694-3.540	<0.001	0.484	0.247-0.946	0.034
PF+ CRP/Alb						
PF-high+ CRP/Alb-high *vs* PF-high or CRP/Alb-high *vs* PF-low+ CRP/Alb-low	2.528	1.599-3.996	<0.001	8.652	3.562-21.015	<0.001

P-values that achieved statistical significance (p < 0.05) are indicated in bold. Alb, albumin; CRP, C-reactive protein; LMR, lymphocyte-monocyte ratio; LNM, lymph node metastasis; NLR, neutrophil-lymphocyte ratio; PF, plasma fibrinogen; PLR, platelet-lymphocyte ratio.

After adjustments for confounding variables, multivariate analysis revealed that in the entire cohort of 250 patients, CRP/Alb (HR = 0.471; *p* = 0.026), tumour differentiation (HR = 1.745; *p <*0.001), clinical T stage (HR = 1.513; *p* = 0.023), LNM (HR = 1.459; *p* = 0.042), and the combination of PF + CRP/Alb (HR = 8.034; *p* < 0.001) were independent factors significantly linked to OS. Moreover, among the subset of 232 PDAC patients, the independent prognostic factors for OS were CRP/Alb (HR = 0.484; *p* = 0.034), PF (HR = 0.464; *p* = 0.023), clinical T stage (HR = 1.510; *p* = 0.032), LNM (HR = 1.483; *p* = 0.047), and the combined variable PF + CRP/Alb (HR = 8.652; *p* < 0.001).

**Figure 1:**
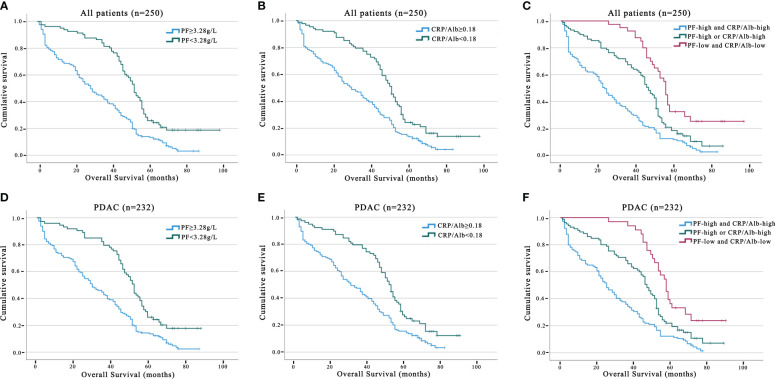
Prognosis value of PF, CRP/Alb in all pancreatic carcinoma and PDAC subgroup patients. The five-year OS rate was determined using the Kaplan–Meier method and assessed with the log-rank test. Analysing OS outcomes associated with PF concentration, including all pancreatic carcinoma patients, and the PDAC subgroup **(A, D)**. Analysing OS outcomes associated with PF concentration, including all pancreatic carcinoma patients, and the PDAC subgroup **(B, E)**. OS analysis involving the combined categorisation based on PF and CRP/Alb for all pancreatic carcinoma patients and the PDAC group **(C, F)**.

We utilised Kaplan−Meier survival curves based on the PF (**Figures 1A, D**) and CRP/Alb (**Figures 1B, E**), and they showed that increased levels of these markers were associated with decreased OS in both the entire group of pancreatic carcinoma patients and specifically in those diagnosed with PDAC. Thus, the patient population was categorised into the following groups: those with elevated and reduced CRP/Alb ratios and those with high and low levels of PF. The five-year OS of patients in the PF level increased group was 12.2%, and that of patients in the PF level decreased group was 17.9%. The five-year OS of patients in the CRP/Alb-increased cohort was 13.6%, and that of patients in the CRP/Alb-decreased cohort was 17.3%.

### 3.3 Association of CRP/Alb and PF with clinicopathological features of pancreatic carcinoma

The correlation between clinicopathologic factors and CRP/Alb and PF was analysed by the Chi-square test. Among the 250 patients with pancreatic carcinoma, advanced clinical T stage (*p* < 0.001), tumour differentiation (*p* = 0.023), and LNM (*p* = 0.005) exhibited significant correlations with high PF levels (≥ 3.28 g/L); advanced clinical T stage (*p* < 0.001), tumour differentiation (*p* = 0.047), and LNM (*p* = 0.002) exhibited significant correlations with high CRP/Alb levels (≥ 0.18) (**Table 3**).

**Table 3:** Clinicopathological features of 250 pancreatic carcinoma patients stratified by PF and CRP/Alb.

Characteristic	Total n = 250 (%)	PF			CRP/Alb		
		≥3.28g/L	<3.28g/L	*p*-value	≥0.18	<0.18	*P*-value
Age (years, n (%)							
<50	93	65	28	0.551	58	35	0.535
≥50	157	104	53		104	53	
Gender, n (%)							
Male	145	100	45	0.588	99	46	0.176
Female	105	69	36		63	42	
Clinical T stage, n (%)							
≤T2	112	61	51	<0.001	58	54	<0.001
>T2	138	108	30		104	34	
Tumour differentiation, n (%)							
Well/moderately	104	62	42	0.023	60	44	0.047
Poorly	146	107	39		102	44	
Tumour size, n (%)							
<2cm	104	68	36	0.528	66	38	0.708
≥2cm	146	101	45		96	50	
LNM, n (%)							
No	101	58	43	0.005	54	47	0.002
Yes	149	111	38		108	41	

P-values that achieved statistical significance (p < 0.05) are indicated in bold. Alb, albumin; CRP, C-reactive protein; LNM, lymph node metastasis; PF, plasma fibrinogen.

Among the 232 patients with PADC, advanced clinical T stage (*p* < 0.001), tumour differentiation (*p* = 0.048), and LNM (*p* = 0.033) exhibited significant correlations with high PF levels (≥ 3.28 g/L). In addition, advanced clinical T stage (*p* < 0.001), tumour differentiation (*p* = 0.024), and LNM (*p* < 0.001) exhibited significant correlations with high CRP/Alb levels (≥ 0.18) (**Table 4**).

**Table 4:** Clinicopathological features of 232 PDAC patients stratified by PF and CRP/Alb.

Characteristic	Total n = 232 (%)	PF			CRP/Alb		
		≥3.28g/L	<3.28g/L	*p*-value	≥0.18	<0.18	*P*-value
Age (years, n (%)							
<50	86	61	25	0.526	54	32	0.671
≥50	146	232	48		96	50	
Gender, n (%)							
Male	135	95	40	0.478	92	43	0.211
Female	97	64	33		58	39	
Clinical T stage, n (%)							
≤T2	106	58	48	<0.001	55	51	<0.001
>T2	126	101	25		95	31	
Tumour differentiation, n (%)							
Well/moderately	96	59	37	0.048	54	42	0.024
Poorly	136	100	36		96	40	
Tumour size, n (%)							
<2cm	94	63	31	0.774	59	35	0.675
≥2cm	138	96	42		91	47	
LNM, n (%)							
No	88	53	35	0.033	44	44	<0.001
Yes	144	106	38		106	38	
CRP/Alb							
≥0.18	150	113	37	0.003			
<0.18	82	46	36				

P-values that achieved statistical significance (p < 0.05) are indicated in bold. Alb, albumin; CRP, C-reactive protein; LNM, lymph node metastasis; PF, plasma fibrinogen.

### 3.4 The prognostic relevance of the CRP/Alb and PF combined indices in 250 patients with pancreatic carcinoma

**Figure 2:**
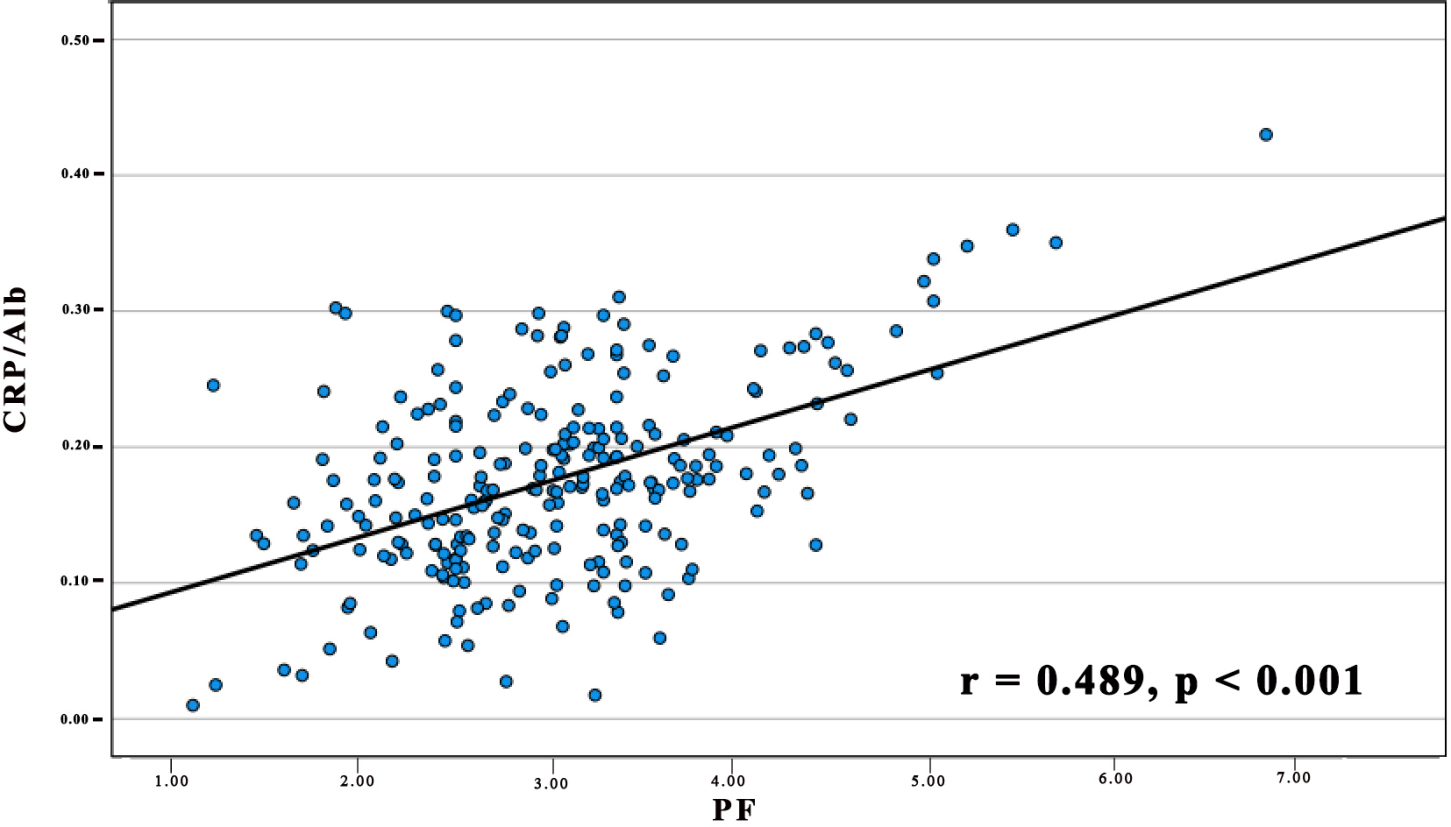
The relationship between PF and the CRP/Alb Ratio in 250 Patients diagnosed with pancreatic carcinoma. The preoperative PF displayed a positive association with the CRP/Alb ratio. (r = 0.489, *p* < 0.001).

We further analysed the link between PF and the CRP/Alb ratio utilising Spearman’s test (**Figure 2**). The findings revealed a positive correlation between the CRP/Alb ratio and PF (r = 0.489, p < 0.001) in all cases of pancreatic carcinoma. Combining the CRP/Alb ratio with the PF could enhance patient stratification by OS (**Figures 1C, F**). Consequently, we categorised patients into three cohorts: (I) those with both low PF and low CRP/Alb, (II) those with either high PF or high CRP/Alb, and (III) those with both high PF and high CRP/Alb. These categories aligned with low, moderate, and high-risk cohorts, respectively. The 5-year OS rates for the 250 patients were 27.8%, 19.5%, and 13.2%, respectively, while for the 232 PDAC patients, the 5-year OS rates were 27.6%, 18.8%, and 12.9%, respectively. Multivariate analysis was also conducted, and as outlined in **Table 1**, it became evident that patients in both the high-risk and moderate-risk cohorts experienced notably worse prognoses than patients in the low-risk cohorts.

## 4 Discussion

Pancreatic carcinoma is complex and heterogeneous, leading to varying rates of recurrence and progression (22). With an enhanced comprehension of the molecular biology of pancreatic carcinoma, advancements in the diagnosis and treatment of this invasive disease have been achieved. Previous conclusive evidence has shown that the tumour-related inflammatory system is essential for tumour angiogenesis and tumorigenesis and that two parameters (fibrinogen and CRP/Alb) of inflammation-based biomarkers may be associated with the prognosis of pancreatic carcinoma (23–25). PF and CRP/Alb have been correlated with clinical outcomes in many types of tumours (20, 26).

The present study demonstrated the following: (1) pancreatic carcinoma patients with high PF (> 3.28 g/L) and CRP/Alb (> 0.18) levels had lower survival rates; (2) pancreatic carcinoma patients with high PF and CRP/Alb levels had higher tumour grades and clinical stages and were more prone to lymph node metastasis; (3) the levels of PF and CRP/Alb are unrelated to the age, gender, or tumour size of pancreatic cancer patients; (4) A noteworthy association was observed between increased PF levels and higher CRP/Alb values in individuals diagnosed with pancreatic carcinoma; (5) The predictive power of PF for OS was specific to PADC patients and did not extend uniformly to all pancreatic carcinoma cases; and (6) combining PF and CRP/Alb levels has the potential to enhance the accuracy of predicting survival outcomes in pancreatic carcinoma patients. These study results provide unique insights into individual research findings, with a focus on the hypothesis that PF and the CRP/Alb ratio are prognostic factors for pancreatic cancer. We suggest that additional adjuvant therapy may be beneficial for high-risk patients. While these conclusions require further validation, the data offer new insights into the biological invasiveness of pancreatic carcinoma in the Chinese population.

The prognostic importance of the PF level in pancreatic carcinoma can be attributed to its underlying biological mechanisms. As a vital constituent of the coagulation/fibrinolytic system, fibrinogen is a pivotal acute-phase protein, and its plasma levels are significantly influenced by inflammation (27–29). Research has indicated that fibronectin interacts with various growth factors, such as those involved with its role in suppressing cell apoptosis and its interaction with members of the PDGF family. These interactions subsequently lead to tumour cell adhesion, the production of VEGF, metastasis, and the activation of the FGF family (30, 31). In addition, cell line models have demonstrated that fibronectin can induce EMT through the p-AKT/p-mTOR pathway to promote cancer cell motility (32). Within a research investigation involving 211 individuals diagnosed with pancreatic carcinoma, Qi et al. confirmed that the hyperfibrinogen concentration could be a potential factor for evaluating the accuracy of OS prediction (33). In addition, a hyperfibrinogen concentration ≥400 mg/dL was found to be an unfavourable predictor of PFS and OS in patients with pancreatic carcinoma treated with chemoradiotherapy (34). Based on these studies, we suggest that the PF concentration in pancreatic cancer patients can be considered an adverse prognostic marker and tends to indicate a poor prognosis.

In addition, our univariate analysis demonstrated that among 250 pancreatic cancer patients and even within the subset of 232 PDAC patients, an elevated NLR was associated with a shorter OS. This evidence enhances our understanding of the link between chronic inflammation and survival in individuals with pancreatic carcinoma. An elevated NLR predicts a more adverse prognosis for pancreatic carcinoma patients, consistent with the findings of a survey involving 263 pancreatic carcinoma patients (35). The potential biological mechanism of the NLR effect stems from the infiltration of neutrophils and lymphocytes. The chronic inflammatory response induced by tumour cells further enhances the infiltration of neutrophils, further promoting tumour progression through the secretion of interleukin-2 (IL-2), IL-6, IL-10, tumour necrosis factor-alpha (TNF-α), and vascular endothelial growth factor (VEGF) (36, 37). VEGF, known for its role as an oncogene, is acknowledged to enhance cell migration by increasing vascular permeability. Furthermore, elevated levels of TNF-α and IL-10 induce a decrease in the number of lymphocytes and the occurrence of functional impairments (38, 39).

As an acute-phase protein during inflammation, CRP is induced by and synthesised in the liver through proinflammatory cytokines. It is considered a predictive factor for systemic infections (40). Albumin is a circulating protein present in the plasma that is directly involved in the occurrence of inflammation (41). CRP/Alb is a combined index of the serum ALB concentration and total CRP concentration. It was identified as one of the prognostic indicators for sepsis patients and has subsequently been recognised as a prognostic indicator for tumours as well (13, 42). For instance, researchers have shown that, in the assessment of tumour prognosis, The predictive utility of the CRP/Alb ratio is comparable to that of the mGPS. The CRP/Alb ratio may exhibit greater predictive accuracy than the mGPS, possibly due to the theoretical advantage of better utilising the CRP and Alb levels in the CRP/Alb ratio (43). Liu et al. reported that the independent prognostic capability of the CRP/Alb ratio is noteworthy in individuals with stage III and IV pancreatic carcinoma, but evidently not in patients with pancreatic cancer clinically staged as stage I or II (44). The research by Fan et al. substantiated the CRP/albumin ratio’s role as a tool for predicting survival rates and gauging the effectiveness of chemotherapy in advanced pancreatic carcinoma (45). Based on the cutoff value (> 0.18) used, our research findings indicate that among 250 pancreatic cancer patients, even within the PADC subgroup, an elevated CRP/Alb ratio signifies a shorter OS. Therefore, the CRP/Alb ratio demonstrates promise as an inflammatory marker, bearing significance for the clinical prognosis of those with pancreatic carcinoma.

Considering both PF levels and CRP/Alb ratios together, we found that patients in the high PF cohort and high CRP/Alb cohort exhibited the lowest survival time. This observation further underscores the connection between high CRP/Alb and PF levels and the inflammatory response in patients with pancreatic carcinoma that ultimately leads to an unfavourable outcome. Both CRP and fibrinogen are critical factors that occur during the acute phase response to inflammation and serve as key acute-phase proteins (46, 47). Activation of the coagulation system is widely acknowledged to be initiated by inflammation, resulting in an increase in prothrombin and antifibrinolytic factor levels (48, 49). In contrast, elements within the coagulation system, including fibrinogen or fibrin, can independently trigger the onset of the inflammatory process and contribute to the development of subsequent tissue damage downstream (50). Therefore, fibrinogen can be considered an important driving factor in the occurrence of chronic inflammation. Our observation of a robust association between PF and the CRP/Alb ratio highlights the intricate interplay between the coagulation/fibrinolytic system and the inflammatory tumour microenvironment and suggests its potential to impact patient survival (51, 52).

This research is not without limitations, and the primary concern is the retrospective design, which brings the potential for selection bias into consideration. Second, the methods employed to measure PF and the CRP/Alb ratio were not uniform, leading to variations in the optimal cutoff values. Third, potential biases may have arisen from clinical factors, such as ethnicity and age.

In summary, our research suggested that the PF concentration was an accurate, convenient, inexpensive biomarker for pancreatic carcinoma, particularly in the case of PDAC patients. The evaluation of PF and the CRP/Alb ratio may therefore be informative for deciding surgical strategies. Furthermore, the combination of the CRP/Alb ratio and the PF could increase the precision of prognosis prediction in pancreatic carcinoma patients.

## Data availability statement

The original contributions presented in the study are included in the article/**Supplementary Material**. Further inquiries can be directed to the corresponding author.

## Ethics statement

The studies involving humans were approved by Medical Ethics Committee of Longyan Second People’s Hospital (LYEYEC 2023-025). The studies were conducted in accordance with the local legislation and institutional requirements. The participants provided their written informed consent to participate in this study.

## Author contributions

XC: Writing – original draft, Writing – review & editing. ZC: Writing – original draft, Data curation, Formal analysis. JG: Data curation, Writing – original draft. ZX: Data curation, Formal analysis, Writing – review & editing. HC: Writing – original draft.

## Supplementary material

Supplementary Figure 1 The ROC curves of PF **(A)** and CRP/Alb **(B)** for OS in pancreatic carcinoma patients.
