# Supplementary material for: Preoperative plasma fibrinogen and C-reactive protein/albumin ratio as prognostic biomarkers for pancreatic carcinoma
Source: Front Oncol. 2024 Mar 1;14:1301059. doi: 10.3389/fonc.2024.1301059 (PMC10943689; doi:10.3389/fonc.2024.1301059)
Supplement: Supplementary Figure 1 — The ROC curves of PF (A) and CRP/Alb (B) for OS in pancreatic carcinoma patients. [file Image_1.pdf]

Supplementary Figure 1. The ROC curves of PF (A) and CRP/Alb (B) for OS in pancreatic carcinoma patients.

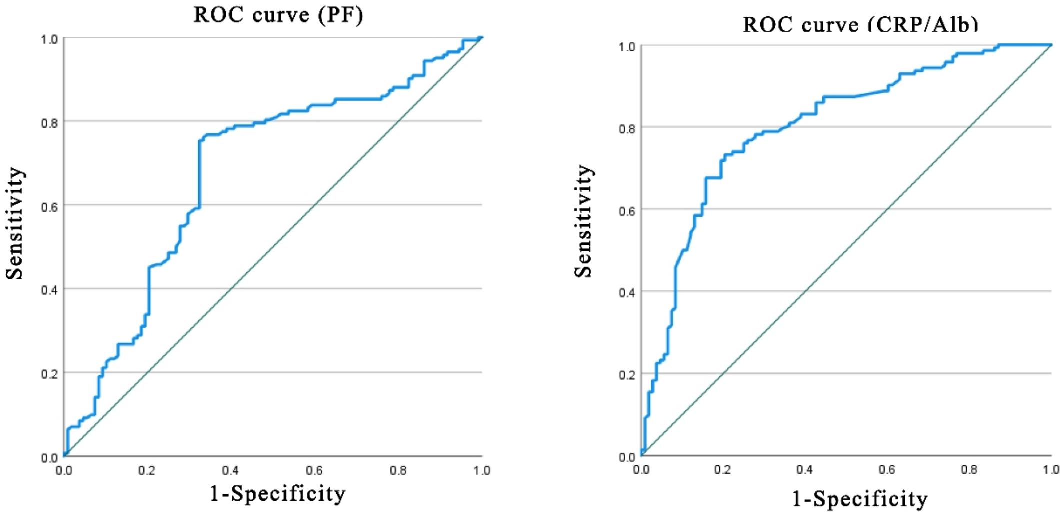

Variable	AUC	SE	P-value	95% CI
PF	0.679	0.035	<0.001	0.610-0.748
CRP/Alb	0.803	0.028	<0.001	0.747-0.858
